# Diagnosis of Felty’s syndrome, distinguished from hematological neoplasm: A case report

**DOI:** 10.3892/ol.2013.1779

**Published:** 2013-12-27

**Authors:** RUO-ZHI XIAO, MU-JUN XIONG, ZI-JIE LONG, RUI-FANG FAN, DONG-JUN LIN

**Affiliations:** 1 Department of Hematology, Third Affiliated Hospital, Sun Yat-Sen University, Guangzhou, Guangdong 510630, P.R. China; 2 Sun Yat-Sen Institute of Hematology, Sun Yat-Sen University, Guangzhou, Guangdong 510630, P.R. China

**Keywords:** Felty’s syndrome, diagnosis, treatment

## Abstract

Felty’s syndrome (FS) is characterized by the three conditions of rheumatoid arthritis (RA), neutropenia and splenomegaly, and occurs in few cases of longstanding erosive RA. Discriminating between rare occurrences of autoimmune diseases and malignancies is crucial. The present study describes the case of a 17-year-old female with a two-year history of RA, presenting with an irregular fever, hepatosplenomegaly and enlarged lymph nodes. The antinuclear antibody titer was 1:320, while antibody results for anti-dsDNA, anti-Sm and rheumatoid factor were negative. The clinical presentation was similar to that of lymphoma. However, the fluorodeoxyglucose-positron emission tomography and biopsy examinations of the liver and cervical lymph node did not support the diagnosis of lymphoma. According to the laboratory results and clinical symptoms, the differential diagnosis indicated FS, and immunosuppressive agents were administered. Two weeks later, the patient no longer had a fever, and the transaminase levels were normal, associated with shrinkage of the liver and spleen.

## Introduction

Felty’s syndrome (FS), which was first described in 1924, is a specific subcategory of rheumatoid arthritis (RA) characterized by the triad of RA, severe extra-articular disease and unexplained neutropenia (1). FS usually develops after a >10-year course of RA and accounts for <1% of RA patients (2). The exact cause is unknown, however, several risk factors have been proposed, including autoimmunity. The active extra-joint clinical features can be misleading in FS and certain clinicians focus on the severe extra-articular disease and neutropenia, which may lead to subsequent infections. Thus, the correct diagnosis is occasionally challenging.

The present study describes a case of FS with an atypical arthritis presentation. The patient presented with a fever, enlarged lymph nodes and hepatosplenomegaly, which can easily be misdiagnosed as a hematological neoplasm. Written informed consent was obtained from the patient.

## Case report

A 17-year-old female with a 2-year history of erosive nodular seropositive RA followed Chinese herbal treatment for symptoms of repeated arthralgia in 2011. One year later, the patient developed jaundice, hepatosplenomegaly and enlarged lymph nodes. A physical examination revealed deformity in the proximal interphalangeal joints, metacarpophalangeal joints, wrists and ankles. Lymph nodes at the posterior neck, axillary fossa and inguinal area were enlarged with a diameter of 0.5–1 cm.

**Figure 1:**
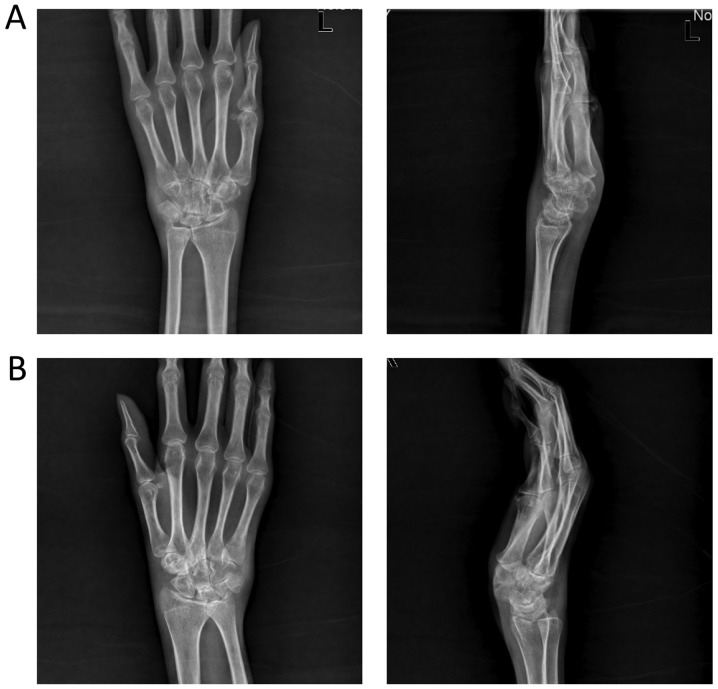
Joint X-rays of (A) left and (B) right wrists.

**Figure 2:**
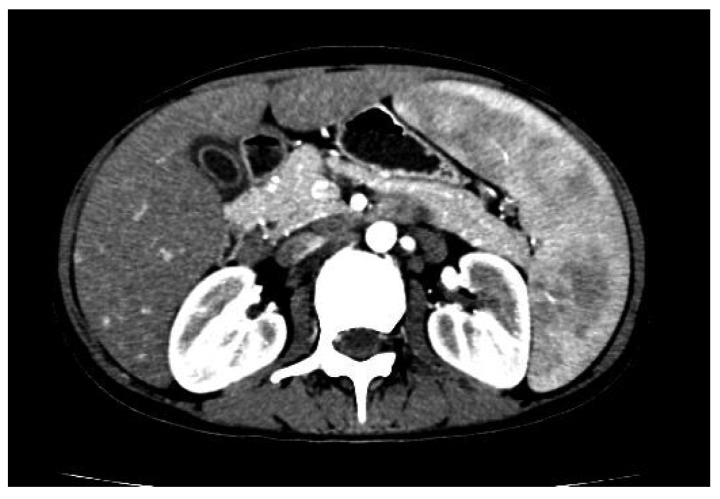
Abdominal computed tomography (CT) scan of the patient with Felty’s syndrome (FS).

**Figure 3:**
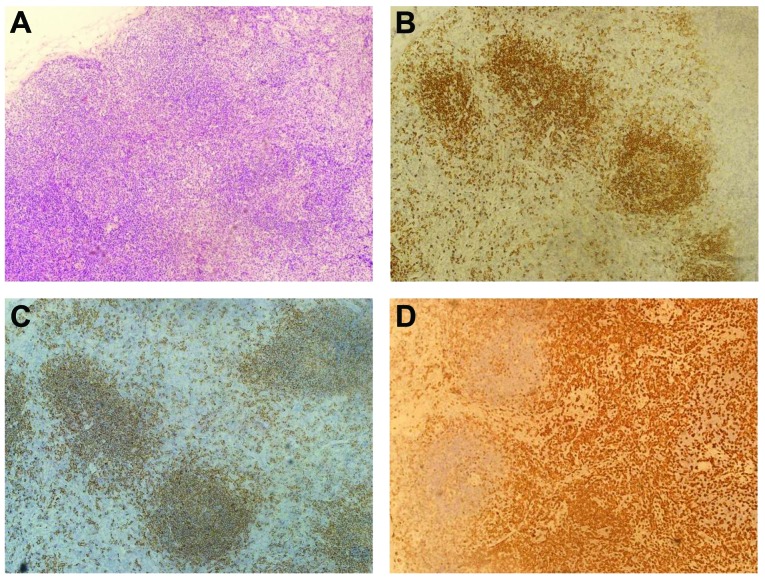
Microscopic analysis of the right cervical lymph node. (A) Hematoxylin and eosin staining. (B–D) Immunohistochemical staining. Positive staining for (B) cluster of differentiation (CD)79α and (C) CD20 in interfollicular regions. (D) Positive staining for CD5 in the paracortical area. Magnification, ×100.

**Figure 4:**
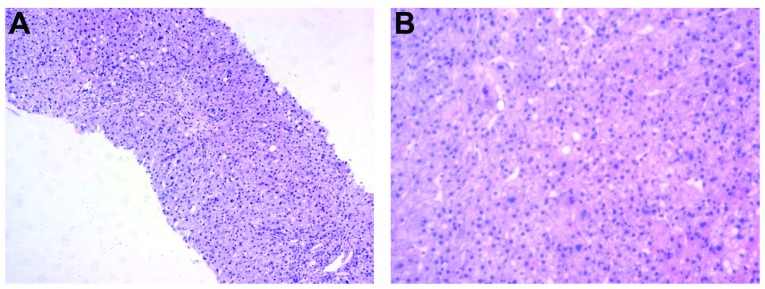
Pathological evaluation of liver tissue by H&E staining. (A) Magnification, ×100. (B) Magnification, ×200.

In August 2012, the patient presented with a fever >39°C. Blood tests revealed a white blood cell count of 4.12×10^9^/l with an absolute neutrophil count of 3.15×10^9^/l, hemoglobin levels of 80 g/l and a platelet count of 159×10^9^/l. A bone marrow biopsy showed normocellular and maturing trilineage hematopoiesis. Glutamic oxaloacetic transaminase levels were 352 U/l, while glutamate pyruvate transaminase levels were 136 U/l. Bilirubin levels were 112.8 μmol/l and lactate dehydrogenase levels were 548 U/l. A rapid erythrocyte sedimentation rate (35 mm/h) and high C-reactive protein levels (60.2 mg/l) were revealed. Cultures of blood and secreta showed no infection by microorganisms. Tests for human immuodeficiency virus, syphilis, hepatitis B, hepatitis C and autoimmune hepatitis-associated antibodies, and a purified protein derivative skin test produced negative findings. The antinuclear antibody titer was 1:320, while results for anti-dsDNA, anti-Sm and ant-Rnp antibodies were negative. Levels of complement C3 and C4 were normal. Rheumatoid factor, antibodies to cyclic citrullinated peptides, anti-keratin antibodies and anti-perinuclear factor autoantibodies were negative. X-rays of the joints, including the wrists and hands, showed soft-tissue swelling, bone erosion and narrowing of the joint cavity (Fig. 1). Splenomegaly (54×124 mm) with uniform density and multiple enlarged lymph nodes distributed along the retroperitoneal space, omental bursa, mesentery root and surrounding hepatic hilar was shown by abdominal computed tomography (CT) (Fig. 2). A lymph node biopsy from the right cervical node revealed chronic inflammation, with positive results for cluster of differentiation (CD)79α and CD20 in interfollicular regions, and for CD5 in the paracortical area (Fig. 3). Fluorodeoxyglucose (FDG)-positron emission tomography revealed lymphadenectasis of the bilateral submandibular and superficial anterior cervical lymph node, axillary fossa, retroperitoneal space, pelvic wall and inguinal area without FDG uptake. The proliferation of hypermetabolic lesions was observed in the shoulder, hip, knee, ankle and interphalangeal joints, as well as knee joint-effusion, which was the manifestation of RA. FDG uptake in the liver and spleen occurred with a maximal standardized uptake value of 1.4 and 2.0, respectively. The pathological evaluation of liver tissue showed inflammatory infiltration containing swelling and spotty or fragmented necrosis in the hepatocytes (Fig. 4). Based on these findings, the provisional diagnosis was of FS.

Treatment was initiated with methotrexate (7.5 mg/week), hydroxychloroquine (400 mg/day) and methylprednisolone (16 mg/day). After tolerating overall treatment, the patient did no longer had a fever. The levels of liver-associated enzymes and bilirubin, and the erythrocyte sedimentation rate were decreased. This decrease is usually associated with liver and spleen shrinkage. Long-term follow-up is currently being performed.

## Discussion

Severe joint destruction contrasting with moderate or absent joint inflammation and severe extra-articular disease, including, hepatopathy, lymphadenopathy, vasculitis, leg ulcers, abnormal skin pigmentation and a high frequency of rheumatoid nodules, are the clinical characteristics of FS. Due to severe neutropenia, the affected individuals are susceptible to skin ulcer formation and sepsis (2). There is no specific diagnostic criterion for FS. FS is a clinical diagnosis in patients with RA with unexplained neutropenia and splenomegaly. Occasionally, the inactive joint symptoms lead clinicians to focus on the severe extra-articular disease and neutropenia, subsequently causing recurrent and even fatal infections. Therefore, the correct diagnosis is challenging, and FS can easily be misdiagnosed as a hematological neoplasm.

The hematological manifestations of RA can be conveniently categorized into the following broad areas: Anemia related to non-steroidal anitflammatory drugs or chronic disease, FS or large granular lymphocyte syndrome-induced neutropenia and hematological malignancy. Hepatosplenomegaly, neutropenia and thrombocytopenia, often indicative of the diffuse spread of a malignancy, may occasionally signify an underlying benign inflammatory process in association with localized lymphoma. Patients with long-standing FS disorders are considered at higher risk of developing lymphoma (3). An association exists between large granular lymphocyte (LGL) leukemia and monoclonal expansion of LGLs and infiltration of the bone marrow and spleen, which has a similar clinical presentation to FS; this has also been termed ‘pseudo-Felty’ (4). The pivotal T cells involved in RA pathogenesis have biological properties that are remarkably similar to those in LGL leukemia (5). Liu and Loughran (6) found that there is an expansion of LGLs in 30–40% of patients with FS, and indicated that FS and LGL leukemia with RA may be part of the same disease spectrum, due to the prevalence of the immunogenic marker HLA-DR4 in the two diseases. RA-associated LGL leukemia, lymphoma and clinical manifestations of typical FS are occasionally not distinguishable; therefore, the correct diagnosis of FS is particularly important.

Neutropenia is the most common finding in FS. A previous study showed that after mice were injected with the serum from patients with FS, the number of circulating polymorphonuclear cells decreased and human immune complexes were deposited in the vascular bed of the lungs, which accounted for the pathogenesis of the neutropenia in FS (7). Dwivedi *et al* (8) proposed that FS autoantibodies bind to deiminated histones and neutrophil extracellular chromatin traps, which further stimulates neutrophils, thus completing a self-sustaining cycle that drives the depletion of mature neutrophils. Moreover, a long course of erosive RA has also been indicated to contribute to FS neutropenia as a consequence of immune complex-driven neutrophil depletion (9). In the present case, the patient had only a two-year history of RA, therefore the number of neutrophils was normal.

The majority of experts recommend that the initial choice of treatment for FS with increased infection rate should be low-dose methotrexate (MTX) (1). Other disease-modifying anti-rheumatic drugs (DMARDs), including hydroxychloroquine, cyclosporin A, sulfasalazine, leflunomide, azathioprine and cyclophosphamide, have also been used in the treatment of FS (10). Rituximab is used as a second-line therapy in patients with refractory FS who are unresponsive to DMARDs (11,12). Other anti-TNFα agents, including etanercept, infliximab and adalimumab, have been reported as new methods to treat FS, while anti-TNFα in combination with colony-stimulating factors (granulocyte colony-stimulating factor) may be used with a potential reduction of the infectious risk. In the present case, the leukocyte and neutrophilic granulocyte counts were normal. By two weeks of treatment with MTX, hydroxychloroquine and methylprednisolone, the patient was in symptomatic remission and long-term follow-up is currently being performed.
